# Supplementary material for: Children’s and Adolescents’ Use of Nature During the COVID-19 Pandemic in a Very Green Country
Source: Int J Environ Res Public Health. 2024 Nov 18;21(11):1530. doi: 10.3390/ijerph21111530 (PMC11593491; doi:10.3390/ijerph21111530)
Supplement: Supplementary file 1 [file ijerph-21-01530-s001.zip › Table S1.pdf]

Table S1. Questionnaire form for the survey

The original survey questionnaire was in Norwegian. This English version has been translated by the authors for an international readership.

To all – if you have more than one children, answer on behalf of the oldest child.

1. **About the child**

a) How old is the child?
Dropdown (6–19 years)

b) Is the child a boy or girl?
Boy
Girl

c) Is the child living in?
One home
More than one home
Has moved away from home

d) How many children live in the household?
Dropdown

e) Where does the child live?
City with more than 50,000 inhabitants
City with between 5000 and 50,000
Village with between 200 and 5000
Rural area

2. **Is the child a member of any of these outdoor life organizations today, or has he/she been a member in the past?**

	Participating now	Participated previously
Norwegian Guide and Scout Association		
4H		
DNT – Norwegian Trekking Association (DNT ung (young))		
Norwegian Association of Hunters and Anglers		
Norwegian Ski Federation		
Red Cross		
Sports association that makes use of nature in whole or in part		
Other team/group related to outdoor activities (e.g. climbing, dog sledding, cycling, orienteering, sailing, rowing, paddling, hiking)		

☐ No, the child has not taken part in any of these organizations.

3. **Is the child a member of any of these outdoor life organizations today, or has he/she been a member in the past?**

	Participating now	Participated previously
Sports team/sports club		
The cultural school		
Band/choir/orchestra/music club		

Theatre/dance		
Environmental agents or other environmental organisation		
Other organized activity – which one?_____________________		

☐ No, the child has not taken part in any of these organizations.

Those who are now part of one or more outdoor life organizations (in question 2) are asked these questions (those who may have children who are both part of and have also left an organization are asked the questions in the green box only, not the those in blue box).

4. **If the child is part of several outdoor life organizations, which of these has the child been part of the longest? (if the child is only in one organization, state this).**

Norwegian Guide and Scout Association
4H
DNT – Norwegian Trekking Association (DNT ung (young))
Norwegian Association of Hunters and Anglers
Norwegian Ski Federation
Red Cross
Sports association that makes use of nature in whole or in part
Other, which? ______________________________

5. **When did the child start in this organization?**

Dropdown 2020–2005

6. **Roughly how often does the child participate in activities run by this organization (answer for the season if the activity is seasonal)?**

5 times per month or more often
4 times per month/once per week
3 times per month
1–2 times per month
More rarely

7. **Was it primarily you (the adult) or the child who suggested that the child should start in this organization?**

The child
I/we adult(s)

8. **Where did the child or you as a parent(s) become aware of this organization? (you can tick several options)**

From friends
Someone in the family/we as parents mentioned it to the children
From school or preschool
From information from the organization's webpage/Facebook page, etc.
From media (newspaper, magazine etc.)
From brochure *or*
via information/advertisements in stores, etc.
Directly from the organization through an information meeting, written information, etc.)
I/we have always known about the offer (and sought information about registration myself/ourselves)
Do not remember

9. 9. How important were the following reasons for the decision that the child should start in this organisation? *Response scale: 1 not important at all to 7 very important*

The child wanted to
The child does not thrive in organized sports
The child gets to be outside a lot
The child gets to experience nature
The child had friends who were there
The child gets to learn practical outdoor life skills

The child gets the opportunity to play a lot in nature
I/we thought it was important that the child got to know the connections in nature
I/we thought that the child would have the opportunity to learn about nature/outdoor life
I/we thought it would be good for the child's development
I/we are active in the outdoors in my/our spare time
I/we are members of an outdoor life organization myself/ourselves

10. **Are there other reasons that were important?**
Open-ended question

11. **How good/bad do you think your child thinks it is to be part of this organization?**
Response scale: 1 not important at all to 7 very important

12. **How good/bad do you think the offer from this organization is?**
Response scale: 1 very bad at all to 7 very good

Those who are no longer part of one of the outdoor life organizations (in question 2) are asked these questions. (note that if they ***are participating in one organization****, they should not be asked these questions, even if they have joined one or more others).*

13. **If the child has joined several outdoor life organizations, which of these did the child join last? (if the child has only left one organization, state this).**
Open-ended question

14. How old was the child when he/she started and left this organization?
Dropdown years

15. **Who decided that the child should leave this organization?**
The child himself/herself
I/we adult(s)
The child and adults together

16. If you think back to when the child was with this organization, how good/bad do you think your child thought it was to participate?
Response scale: 1 very bad at all to 7 very good

How good/bad do you think the offer was?
Response scale: 1 very bad to 7 very good

17. How important were the reasons mentioned below for the child leaving this organization?
Response scale: 1 not important, 2 slightly important, 3 very important

- It took too much time
- The child lost interest/didn't want to participate anymore
- Prioritizing school work
- It was too expensive
- The child did not get much out of it socially
- Friends stopped doing the activities
- The management/organization of the offer was too poor
- Demand for a lot of equipment/expensive equipment
- The child's health
- We moved
- The offer/organisation's activities ceased
- Other activities were more interesting – which ones?
- Other, what?___________

18. **Is there anything that could have caused the child to continue to be part of this organization?**
Open-ended question

Those who have never been part of any of the outdoor life organizations are asked these questions (question 2)

An estimated 10–12% of all children/adolescents have joined an outdoor life organization (4H, Norwegian Guide and Scout Association, Norwegian Trekking Association, Norwegian Ski Federation, Norwegian Association of Hunters and Anglers, and other groups/organizations that operate with/nature-based outdoor activities). We want to find out more about the reasons why participation is not higher. We list several imaginary preconceptions about why your child has not been involved in any of the outdoor life organizations. Indicate how well you think the statements fit you/you and your child.

19. **The child has not been part of an outdoor life organization because ...**
Response scale: 1 sounds very bad 7 sounds good
…I/we are not aware of any activity under the auspices of outdoor life organizations
...The child is not particularly interested in outdoor activities
...The child thrives better with unorganized activity
...The child is not too happy to be outside/enjoys indoor activities better
...I/we adult(s) think it is too expensive to participate
...The child has no friends who are involved in outdoor life organizations
...There are no such offers near where we live
…It requires too much driving/other assistance from us adults
...Requirements for volunteer efforts for parents/caregivers`
...We/the child do/does not have time to be involved
...The child has never expressed a desire to be involved
...I/we adults am/are not interested in outdoor life, hunting, fishing, or use of nature
...I/we adults think school work is more important
...I/we adults think cultural activities are more important
…There is too much bad weather here
...The child does not have the health to participate
…The child is more interested in other organized activities, which:_______________

To all:

20. Has your child ever taken part in a holiday offer (summer, autumn, winter) for which outdoor activities/being out in nature were an important part of the offer?
Yes
No

If yes to question 20:
21. How many times?
Dropdown (1, 2, 3 … 20, more than 20 times)

If yes to question 20:

22. If you think about the times the child/you has/have taken part in such holiday offers
... how good/bad do you think your child think it was?
... how good/bad do you think the offer was?
Response scale: 1 very bad 7 very good

23. Has the child or you as a family taken part in other individual weekend or evening events with a focus on outdoor life/nature (e.g. get-outdoors arrangements, family fishing day, outdoor-life day, etc.)

Yes
No

If yes to question 23:

24. How many times would you estimate that the child has taken part in such events (either alone or together with parents)?
Dropdown (1,2,3 … 50, more than 50 times)

If yes to question 23

25. If you think about the times the child/you has/have taken part in such events
Response scale: 1 very bad 7 very good
... how good/bad do you think your child thinks it was?
... how good/bad do you think the offer was?

Then some questions about outdoor life and the child's/family's use of nature – without this being organized.

26. How do you assess the child's opportunities to develop in natural and green areas close to home? (i.e. nearby environment)
Bad
Quite bad
Neither good nor bad
Quite good
Very good

27. How often does the child tend to play/spend time in natural areas near home either alone or with other children/adolescents?
5 times per month or more often
4 times per month/once a week
3 times per month
1–2 times per month
Less often

28. As a family, do you normally use nearby areas or other natural areas for outdoor activities? If so, how often?
5 times per month or more often
4 times per month/once a week
3 times per month
1–2 times per month
Less often

29. Would you say that you are an 'outdoor family'?
No
Maybe a little
Yes

Then some questions about outdoor activities during the COVID-19 pandemic.

30. What is the child's/adolescent's everyday life like during the pandemic at present time?

	**Less than before the pandemic**	**As usual**	**More than before the pandemic**
They play and stay outdoors in the neighbourhood	☐	☐	☐
They play and stay in natural areas	☐	☐	☐
As a family, we spend time outdoors in natural areas..	☐	☐	☐

31. What is the child's/adolescent's everyday life like in the lockdown period during the pandemic (closed school, preschool, organized activities)?

	**Less than before the lockdown**	**As usual**	**More than before the lockdown**
They play and stay outdoors in the neighbourhood	☐	☐	☐
They play and spend time in natural areas	☐	☐	☐
As a family, we spend time outdoors in natural areas	☐	☐	☐

Question 32 is asked to those who, to question 31, have answered that the child has spent more time than usual either outdoors in the neighbourhood or in natural areas (or both).

32. How often would you estimate that the child played/spent time in natural areas near home alone, with siblings or other children/adolescents when schools/preschools and leisure facilities were closed?
5 times per month or more often
4 times per month/once per week
3 times per month
1–2 times per month
Less often

Question 33 is asked to those who, to question 31, have answered that their child has spent more time than usual either outdoors in the neighbourhood or in natural areas (or both).

33. Who was the child with when more outdoors/in natural areas?
You may choose several options.
alone
with friends
with siblings
with the family
with others, who?____________

To parents whose children/adolescents participated in organized activities.

34. What do you think your child felt about organized activities being closed down during the COVID-19 pandemic?
Response scale: 1 completely disagree to 7 completely agree

Missed meeting friends during organized activities
Missed physical activity in organised activities
Happy to have more leisure time in general
Happy to have more leisure time outdoors
Happy to spend more time with family
Happy to have more time to spend on screen and social media

35. Have you notice anything different today compared to before the first phase of the COVID-19 lockdown in terms of your child's leisure-time activities?
Response scale: 1 completely disagree to 7 completely agree

Spends more time on screens and social media
Spends less time with friends
Spends more time with adults
Stopped doing leisure activities
Less busy weekdays
Engaged in new use of the neighbourhood
Started new hobby/leisure activities

About you who fill out the questionnaire.

36. Are you?
Male
Female

37. How old are you?
Dropdown

38. Which county do you live in?
Dropdown counties

39 What country were you born in?

• Norway

• Other Western European country

• Eastern European country

• Latin American country

• Asian country

• African country

• USA, Canada, Australia, New Zealand

• Don’t know/don’t want to answer

40. What is your daily work?

Multiple choice

 Student

 Full-time employee

 Part-time employee

 Self-employed

 Military service/civil service

 Maternity/parental leave

 Pensioner

 Job seekers

 Stay at home

 Leave of absence

 Social security

 Other

41. Are you the sole parent/caregiver of the child?
Yes
No

42. What is your household’s total gross annual income?
1 Less than 100,000 NOK
2 100–200,000 NOK
3 201-300,000 NOK

4	301–400,000 NOK
5	401–500,000 NOK
6	501–600,000 NOK
7	601–700,000 NOK
8	701–800,000 NOK
9	801–900,000 NOK
10	901–1,000,000 NOK
11	1,001–1,100,000 NOK
12	1,101-1,200,000 NOK
13	1,201–1,300,000 NOK
14	1,301–1,400,000 NOK
15	1,401–1,500,000 NOK
16	More than 1,500,000 NOK
98	Don’t want to answer
99	Don’t know

Thank you for taking the time to answer the survey questions!
