# Supplementary material for: Children’s and Adolescents’ Use of Nature During the COVID-19 Pandemic in a Very Green Country
Source: Int J Environ Res Public Health. 2024 Nov 18;21(11):1530. doi: 10.3390/ijerph21111530 (PMC11593491; doi:10.3390/ijerph21111530)
Supplement: Supplementary file 1 [file ijerph-21-01530-s001.zip › Table S2.pdf]

Table S2. Crosstabs analyses

Table A. Crosstabs analyses to identify relationships between 'Children's play and time spent in neighbourhood during the initial phase of lockdown in the COVID-19 pandemic', and demographic, social, and environmental variables. Significance level 0.05 is indicated in green-shaded cells.

**Variable**	**Label**	**Play and time spent outdoors in neighbourhood during COVID-19 pandemic** (actual/expected value)			**Test statistics** (Pearson's chi-squared, Cramér's V)
		**As usual**	**Less**	**More**	
Child's age (year)	6–8	108 (86)	32 (53)	10 (11)	Pearson's chi-squared = 23.138, df = 6, p < 0.001 Cramér's V = 0.152
	9–12	144 (140)	79 (86)	21 (18)	
	13–15	121 (138)	104 (85)	15 (18)	
	16–19	201 (211)	138 (130)	29 (28)	
Child's gender	Boy	320 (308)	185 (190)	33 (40)	Pearson's chi-squared = 4.05, df = 2, p = 0.132 Cramér's V = 0.064
	Girl	254 (265.8)	168 (166)	42 (35)	
Geographical region	Northern Norway	63 (55)	30 (34)	3 (7)	Pearson's chi-squared = 15.228, df = 10, p = 0.124 Cramér's V = 0.087
	Central Norway	85 (83)	49 (51)	10 (11)	
	Western Norway	115 (115)	67 (71)	18 (15)	
	Eastern Norway	169 (172)	107 (106)	25 (23)	
	Southern Norway (incl. Telemark og Vestfold)	82 (74)	40 (45)	7 (10)	
	Oslo	60 (76)	60 (47)	12 (10)	
Rural vs. urban living (number of inhabitants)	City (> 50.000)	203 (225)	158 (139)	32 (29)	Pearson's chi-squared = 19.303, df = 6, p = 0.004 Cramér's V = 0.098
	Town (5000–50,000	181 (170)	94 (105)	22 (22)	
	Village (200–5000)	119 (124)	81 (76)	17 (16)	
	Rural (< 200)	71 (54)	20 (33)	4 (7)	
Number of homes	One home	461 (445)	269 (277)	51 (59)	Pearson's chi-squared = 9.058, df = 3, p = 0.011 Cramér's V = 0.096
	Several homes	99 (115)	80 (72)	23 (15)	
	Have moved from home	14 (11)	4 (6)	1 (2)	

Number of children in the family	1 child	151 (152)	103 (94)	12 (20)	Pearson's chi-squared = 7.978, df = 4, p = 0.092 Cramér's V = 0.063
	2 children	269 (268)	164 (165)	35 (35)	
	3 or more children	154 (1549	86 (94)	28 (20)	
Member of recreational organized activities	Yes	127 (125)	72 (77)	19 (16)	Pearson's chi-squared = 0.0960, df = 2, p = 0.619 Cramér's V = 0.031
	No	443 (445)	279 (274)	56 (59)	
Parent's gender	Man	298 (275)	156 (169)	26 (36)	Pearson's chi-squared = 10.917, df = 2, p = 0.004 Cramér's V = 0.104
	Woman	276 (299)	197 (184)	49 (39)	
Parent's age (year)	< 30	13 (10)	3 (6)	2 (1)	Pearson's chi-squared = 13.681, df = 6, p = 0.033 Cramér's V = 0.083
	30–39	132 (130)	75 (80)	19 (17)	
	40–49	271 (272)	161 (168)	44 (36)	
	>50	158 (162)	114 (99)	10 (21)	
Annual household income (NOK)	< 499,000	44 (52)	35 (32)	12 (7)	Pearson's chi-squared = 17.404, df = 6, p = 0.008 Cramér's V = 0.100
	500,000 to 999,000	200 (194)	119 (118)	19 (27)	
	1,000,000 to 1,499,000	208 (194)	106 (118)	24 (27)	
	> 1,500,000	44 (56)	41 (34)	13 (8)	
Ethnicity	Norwegian	527 (516)	305 (317)	68 (67)	Pearson's chi-squared = 7.059, df = 2, p = 0.029 Cramér's V = 0.084
	Non-Norwegian	47 (58)	48 (36)	7 (8)	
Caregiver	Sole parent	56 (72)	56 (44)	14 (9)	= 10.154, df = 2, p = 0.006 Cramér's V = 0.101
	2 parents	518 (502)	297 (309)	61 (66)	
Availability of nearby nature	Poor/medium	61 (73)	61 (45)	6 (10)	Pearson's chi-squared = 18.594, df = 4, p < 0.001 Cramér's V = 0.096
	Good	188 (199)	136 (123)	24 (26)	
	Very good	325 (301)	156 (185)	45 (39)	

Table B. Crosstabs analyses to identify relationships between 'Children's play and time spent in natural areas during the initial phase of lockdown in the COVID-19 pandemic', and demographic, social, and environmental variables. Significance level 0.05 is indicated in green-shaded cells.

**Variable**	**Label**	**Play and time spent outdoors in neighbourhood during COVID-19 pandemic** (actual/expected value)			**Test statistics** (Pearson's chi-squared, Cramér's V)
		**as usual**	**(1) less**	**(2) more**	
Child's age (year)	6–8	104 (97)	23 (36)	23 (18)	Pearson's chi-squared = 10.528, df = 6, p = 0.104<br>Cramér's V = 0.072
	9–12	161 (157)	54 (58)	29 (29)	
	13–15	148 (155)	68 (57)	24 (28)	
	16–19	233 (237)	93 (87)	42 (43)	
Child's gender	Boy	352 (347)	126 (128)	60 (63)	Pearson's chi-squared = 0.603, df = 2, p = 0.740<br>Cramér's V = 0.025
	Girl	294 (299)	112 (110)	58 (55)	
Geographical region	Northern Norway	72 (62)	19 (23)	5 (11)	Pearson's chi-squared = 20.441, df = 10, p = 0.025<br>Cramér's V = 0.101
	Central Norway	92 (93)	31 (34)	21 (17)	
	Western Norway	124 (129)	48 (48)	28 (24)	
	Eastern Norway	189 (194)	75 (72)	37 (35)	
	Southern Norway (incl. Telemark og Vestfold)	96 (83)	26 (31)	7 (15)	
	Oslo	73 (85)	39 (31)	20 (16)	
Rural vs. urban living (number of inhabitants)	City (> 50,000)	236 (253)	107 (93)	50 (46)	Pearson's chi-squared = 12.981, df=6, p=0.043<br>Cramér's V = 0.080
	Town (5000–50,000	200 (192)	66 (71)	31 (35)	
	Village (200–5000)	137 (140)	54 (52)	26 (26)	
	Rural (< 200)	73 (61)	11 (23)	11 (11)	
Number of homes	One home	501 (500)	187 (188)	93 (93)	Pearson's chi-squared = 0.059, df = 3, p = 0.971<br>Cramér's V = 0.008
	Several home	128 (129)	50 (49)	24 (24)	
	Have moved from home	12 (13)	5 (5)	2 (1)	
Number of children in the family	1 child	171 (172)	72 (63)	23 (31)	Pearson's chi-squared = 4.840, df=4, p=0.304
	2 children	302 (302)	107 (111)	59 (55)	

	3 or more children	173 (173)	59 (64)	36 (32)	Cramér’s V = 0.049
Member of recreational organized activities	Yes	137 (140)	47 (52)	34 (26)	Pearson’s chi-squared = 3.996, df = 2, p = 0.136<br>Cramér’s V = 0.063
	No	504 (501)	190 (185)	84 (92)	
Parent’s gender	Man	332 (310)	116 (114)	32 (57)	Pearson’s chi-squared = 23.646, df = 2, p < 0.001<br>Cramér’s V = 0.154
	Woman	314 (337)	122 (124)	86 (62)	
Parent’s age (year)	< 30	13 (12)	1 (4)	4 (2)	Pearson’s chi-squared = 23.657, df = 6, p < 0.001<br>Cramér’s V = 0.109
	30–39	147 (146)	46 (54)	33 (27)	
	40–49	309 (307)	103 (113)	64 (56)	
	> 50	177 (182)	88 (67)	17 (33)	
Annual household income (NOK)	< 499,000	52 (59)	25 (21)	14 (11)	Pearson’s chi-squared = 5.722, df = 6, p = 0.455<br>Cramér’s V = 0.058
	500,000 to 999,000	225 (219)	79 (79)	34 (40)	
	1,000,000 to 1,499,000	224 (219)	75 (79)	39 (40)	
	> 1,500,000	60 (64)	22 (23)	16 (12)	
Ethnicity	Norwegian	588 (580)	203 (214)	109 (106)	Pearson’s chi-squared = 7.193, df = 2, p = 0.027<br>Cramér’s V = 0.085
	Non-Norwegian	58 (66)	35 (24)	9 (12)	
Caregiver	Sole parent	73 (81)	34 (30)	19 (15)	Pearson’s chi-squared = 2.923, df = 2, p = 0.232<br>Cramér’s V = 0.054
	2 parents	573 (565)	204 (208)	99 (103)	
Availability of nearby nature	Poor/medium	72 (83)	48 (30)	8 (15)	Pearson’s chi-squared = 24.643, df = 4, p < 0.001<br>Cramér’s V = 0.111
	Good	221 (224)	91 (83)	36 (41)	
	Very good	353 (339)	99 (125)	74 (62)	
